# PCYT2 overexpression induces mitochondrial damage and promotes apoptosis in hepatocellular carcinoma cells

**DOI:** 10.1371/journal.pone.0323974

**Published:** 2025-05-28

**Authors:** Xu Bei, Caihong Zhao, Jia Liu, Jun Cao, Wanbao Zheng, Tao Wang, Wenjie Lu, Youzhi Xu

**Affiliations:** College of Basic Medicine, Anhui Medical University, Hefei, China; Guangdong Medical University, CHINA

## Abstract

Phosphatidylethanolamine cytidyltransferase 2 (PCYT2) is commonly regarded as the rate-limiting enzyme in eukaryotic phosphatidylethanolamine synthesis. However, the role of PCYT2 in the development of hepatocellular carcinoma (HCC) unknow. In this study, the role of PCYT2 overexpression in the development of HCC was examined by culturing HepG2 cells. We compared the expression levels of PCYT2 in L02 cells and HepG2 cells. Then, the HepG2 cells were infected with the lentivirus, establishing PCYT2 overexpression cell models. The proliferation, migration, and apoptotic abilities of PCYT2 overexpression in HepG2 cells was observed using western blotting, CCK-8 assay, Transwell assay, wound healing, and plate cloning methods. Based on this overexpression model, we determined the mitochondrial function and lipid content of HepG2 cells using lipidomics. CDP-ethanolamine (CDP-Etn), a downstream product of PCYT2, was added to the HepG2 cells, inhibiting their proliferation and migration. BALB/c female nude mice inoculated with subcutaneously transplanted tumors were used to explore the role of PCYT2. The results of the in-vitro experiments, shown that the expression of PCYT2 in normal hepatocytes was higher than that in HCC cells, and addition of CDP-Etn and PCYT2 overexpression inhibited the proliferation and migration of HCC cells, promoted the apoptosis of HCC cells, and caused mitochondrial damage. The results of in vivo experiments demonstrated that the tumor volume in the PCYT2 overexpression group was significantly smaller than that in the blank control group. Thus, PCYT2 overexpression inhibits the development of HCC, and its mechanism may be related to the impairment of mitochondrial function.

## Introduction

Hepatocellular carcinoma (HCC) is an exceedingly fatal malignancies, with mortalities that approximate the incidence rates worldwide [1]. HCC is usually diagnosed at advanced stages owing to late symptom manifestations with limited therapeutic options, leading to ineffective intervention and poor prognosis [2]. An increasing number of studies have focused on the progression, pathological features, and prognosis of liver cancer [3–5]. HCC epidemiology is rapidly evolving, one of the most common causes is non-alcoholic fatty liver disease [6], further proving that lipid metabolism plays a crucial role in HCC occurrence. Therefore, the identification of novel therapeutic targets is urgently needed to improve the treatment of patients with HCC [7].

Phosphatidylethanolamine (PE), also known as cephalin, is the most abundant lipid in the cytoplasmic layer of cell membranes and is involved in cellular processes such as membrane fusion [8], autophagy and apoptosis [9–11]. For eukaryotic PE in vivo, two main synthetic pathways exist [12]: including the Kennedy pathway of CDP-ethanolamine(CDP-Etn) and mitochondrial phosphatidylserine decarboxylation pathway [13]. Phosphatidylethanolamine cytidyltransferase 2(PCYT2) is the rate-limiting enzyme of the CDP-Etn pathway. Previous studies have shown that PCYT2 is highly specific, and is present only in the rough endoplasmic reticulum of eukaryotes [14]. P-Eth is then catalyzed by PCYT2 to form CDP-Etn, leading to PE synthesis [15].

Generally, PCYT2 expression is reduced in various epithelial-derived cancer cell lines compared to normal cells [16,17]. Compared epithelial-derived cancer cell lines PCYT2 activity with that of breast epithelial cells MCF-10A showed that its PCYT2 activity was inhibited in breast cancer cells (MCF-7) [16]. PCYT2 expression was significantly reduced in invasive human metastatic colon cancer cells compared to that in primary tumor cells [17], and previous studies have shown that PCYT2 knockdown under nutrient-rich conditions significantly facilitated the proliferation of HeLa and T98G cells and promoted in vivo tumor growth. The inhibition of PCYT2 increases P-Etn levels in cancer cells and stimulates tumor growth [18]. However, in organoid models, PCYT2 knockdown inhibits cell growth [19]. Despite these findings, no evidence exists that suggests that PCYT2 expression is associated with HCC occurrence and development.

This study aimed to investigate the role of PCYT2 in human hepatocellular carcinoma cells (HepG2) by inhibiting their proliferation, invasion, and migration abilities and promoting cell apoptosis.

## Materials and methods

### Materials

CDP-Etn (90756) was purchased from Sigma (Darmstadt, Germany).The ATP assay kit (S0026) and BCA protein assay kit (P0010S) were purchased from Beyotime (Shanghai, China), and RPMI 1640 medium (SH30027.01) was purchased from Gibco (Waltham, MA,USA). Fetal bovine serum(FBS #11012–8611) was purchased from TIANHANG (Zhejiang, China). PCYT2 antibody (ab135290) was provided by Abcam (Cambridge, UK); BAX (#5023), Bcl-2 (#3498), cleaved caspase-3 (#9661) and β-actin (#3700) were purchased from Cell Signaling Technology (Danvers, MA, USA); goat anti-rabbit (E-AB-1034) and goat anti-mouse (E-AB-1035) secondary antibodies were provided by Elabscience (Shanghai, China).

### Cell culture

L02 cells (normal human liver cells) were cultured in RPMI1640 medium, and HepG2 were cultured in DMEM containing 10% FBS and 1% penicillin-streptomycin. The above two cell types of cells were cultured at 37°C in a humidified incubator atmosphere containing 5% CO_2_.

### Construction of the PCYT2 overexpression model

Lentiviral plasmids overexpressing PCYT2 (LV/In) and negative control (NC/In) were purchased from GENECHEM (Shanghai, China). HepG2 cells were infected with LV/In or NC/In, 48 h later, the cells were screened with DMEM medium containing puromycin (2 µg/mL) for approximately 2 weeks. The expression level of PCYT2 was detected using western blotting after 3–4 generations to confirm PCYT2 expressing up-regulation.

### Western blot

RIPA buffer was used to extract total cellular protein. Protein samples were electrophoresed using 9–11% SDS-PAGE, then transferred to PVDF membranes (Merck, Darmstadt, Germany) that were blocked with 5% skimmed milk in TBST, and incubated overnight utilizing primary antibodies, including anti-PCYT2 (1:250), anti-β-actin (1:3000), anti-BAX (1:1000), and anti-Bcl-2 (1:1000). Subsequently, samples were incubated 1–2 h using an appropriate peroxidase-linked secondary antibodies. Consequently, employing β-actin we normalized the protein levels. Images were visualized using the Chemi-Doc MP system (Bio-Rad).

### Cell viability analysis

HepG2 cells were seeded into 96-well plates and incubated with a Cell Counting kit (CCK-8) solution for 40 min at 37°C. Absorbance was measured at 450 nm using a Spectra MAX M5 microplate spectrophotometer to detect cell viability.

### Migration assay

To determine cell migration, the Transwell assay was performed by adding 500 µL of complete medium to a 24-well plate, then placing the Transwell chamber on the plate. The cell suspension was prepared by adding basal medium and 200 µL (3 × 10^5^ cells/200 µL) to each top chamber. Subsequently, the cells were incubated at 37°C for 1 d. The cells that successfully migrated and attached to the surface of the underlying membrane were fixed with paraformaldehyde and stained with 0.1% crystal violet. Five to six fields of view (original magnification: 200×) were randomly selected for cell counting under a light microscope.

### Invasion experiment

Dilute the Matrigel with basal medium (1:8), lay it flat on a Transwell membrane, and incubate at 37°C for 4 h. The subsequent experimental steps were identical to those used for the migration assay, including cell culture, paraformaldehyde fixation and 0.1% crystal violet staining.

### Scratch-induced migration assays

Two milliliter of cell suspension was added to a six-well plate (3 × 10^5^ cells/well) and cultured at 37°C in a humidified incubator atmosphere at 5% CO_2_; 24 h later, the center of each well was scratched with a 100 µL plastic micropipette and the medium was substituted with basal medium. Each well was imaged under a 200 × light microscope at three randomly selected identical locations after culturing for 24 and 48 h. Finally, we measured cell migration ability by comparing the distance between the edges of each wound at 24 and 48 h.

### Colony formation assays

A cell suspension (2 mL, 5 × 10^2^ cells/mL) was added to each well of a six-well plate and incubated for 2 weeks. When the clones were visible to the naked eye, they were washed twice with phosphate-buffered saline (PBS), and the cells were fixed using –4% paraformaldehyde for 10 min. Paraformaldehyde was washed away and 0.1% crystal violet staining was performed for 15 min. The clones were washed twice with PBS, dried at room temperature and the number of colonies was statistically analyzed.

### ATP detection

Cellular ATP levels were measured using an ATP kit (Beyotime). Sample tubes containing 20 μL of sample or standards were placed in a luminometer (SuPerMax 3100) and rapidly mixed with a micropipette. After > 2 s, relative light unit values were measured.

### Electron microscopy

The tiled cells (1 × 10^6^) were removed, immersed in PBS, and the cell surface was rinsed. Then 2.5% of pre-cooled glutaraldehyde was added to the tiled cells at 4°C, fixed at 4°C for 2 h or overnight, aspirated, and soaked in PBS twice for 10 min each. Finally, ethanol gradient dehydration (30, 50, 70, 80, 90, 100%), critical point drying, coating, and electron microscopy was performed to observe mitochondrial morphology.

### Animal experiments

Five-week-old BALB/c female nude mice were purchased from Jiangsu Jicui Pharmaceutical Biotechnology Co, passed SPF level training and assessment, and were routinely reared using standard SPF conditions. PCYT2 overexpressed HepG2 cells and control cells were subcutaneously injected into the left and right sides of the nude mice (approximately 1 × 10^7^ cells on each side, n = 6 tumors in each group). Following 20–25 d, the tumors achieved a certain size and the micewere anesthetized with isopentane and sacrificed; the tumors were collected for follow-up evaluation. The animal experiments were approved by the Animal Ethics Committee of Anhui Medical University and conducted in accordance with the guidelines for the care and use of laboratory animals.

### Statistical analysis

Data were analyzed using GraphPad Prism software (version 8.0), and the results were expressed as mean ± standard deviation (SD). T-test was performed to determine the significant of differences between two groups. A one-way analysis of variance was used for comparison across groups. Statistical significance was set at P < 0.05.

## Results

### PCYT2 expression in HCC

**Fig 1:**
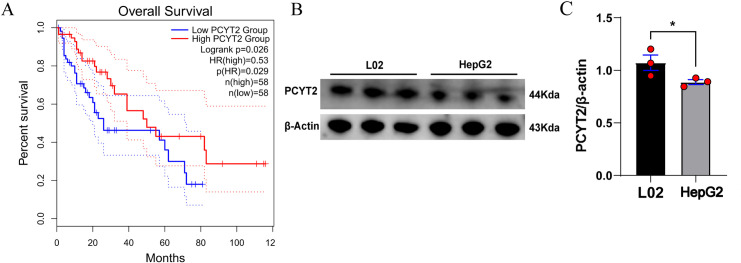
PCYT2 expression in HCC. (A) The relationships between PCYT2 expression and overall survival of patients with HCC. (B–C) The PCYT2 expression level in normal liver cells, L02, and hepatoma cells, HepG2 (n = 3 per group). Data are presented as means ± SD. *P < 0.05.

The Cancer Genome Atlas (TCGA) database, Cancer Research Project, which is a collaboration between the National Cancer Institute and National Human Genome Research Institute, provides a large, free reference database for cancer research by collecting and collating cancer-related data. By analyzing the TCGA database, we attempted to determine the relationship between the PCYT2 expression level and HCC. Our findings demonstrated that overall survival of patients with HCC was closely correlated with PCYT2 expression. We observed that the survival percentage of patients gradually decreased over time; however, patients with high PCYT2 expression levels had a higher survival percentage than those with low PCYT2 expression (Fig 1A). This indicates that high PCYT2 expression levels are beneficial for the overall survival of patients with HCC.

Western blotting was conducted on cultured L02 and HepG2 cells to verify their PCYT2 expression level (Fig 1B and 1C), we found that PCYT2 expression was higher in L02 cells than in HepG2 cells.

### PCYT2 overexpression inhibits HepG2 cell proliferation, invasion and migration and promotes apoptosis

**Fig 2:**
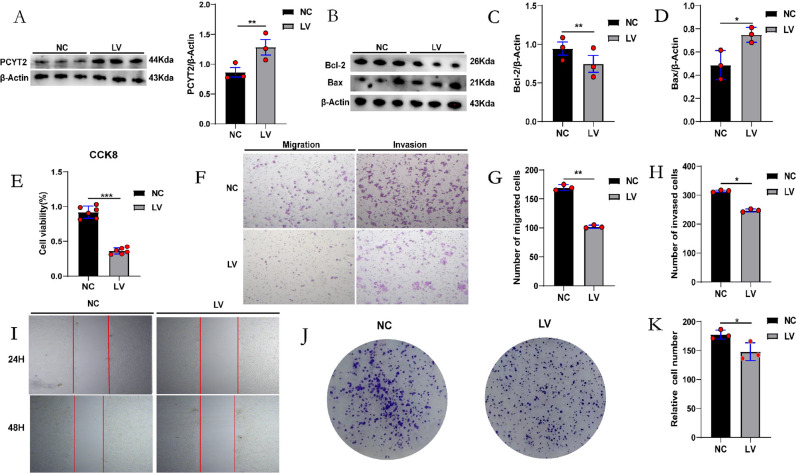
PCYT2 overexpression inhibits HepG2 cell proliferation, invasion, and migration. **(A–D)** The level of PCYT2 (A) as well as the protein expression of Bax and Bcl-2 was measured per group (NC, normal control; LV, lentivirus transfection to over-express PCYT2) using western blotting and representative protein quantification (n = 3 per group). **(E)** The proliferation ability of each group was detected using CCK-8 (n = 6 per group). **(F–H)** A Transwell assay was conducted per group to investigate the effects of cellular migration and invasion. Magnification: 100 × . **(I–K)** The scratch wound healing and colony formation assays of cells post-PCYT2 overexpression. The magnification for scratch wound healing assay is 100 × . Data are presented as means ± SD. *P < 0.05, **P < 0.01, ***P < 0.001.

To verify the effect of PCYT2 on HCC development, we transfected HepG2 cells with a lentivirus and performed western blotting, successfully establishing PCYT2 overexpression in HepG2 cells (Fig 2A). The expression levels of Bcl-2 (an anti-apoptotic factor), Bax (an apoptogenic factor) and cleaved-caspase-3(an apoptogenic factor)(S1 Fig.) were detected using western blotting, and the results revealed that the LV group exhibited increased HepG2 cell apoptosis (Fig 2B–2D). The CCK-8 assay indicated that PCYT2 overexpression significantly reduced HepG2 cells viability (Fig 2E). Utilizing the Transwell assay, we discovered that the HepG2 cells invasion and migratory ability was suppressed post-lentiviral transfection (Fig 2F–2H). The scratch wound healing assay also determined that the migratory ability of the LV group was inhibited (Fig 2I). Additionally, PCYT2 overexpressing HepG2 cells formed less colonies than the negative control group (Fig 2J and 2K). In summary, we demonstrated that PCYT2 overexpression inhibited the proliferation, invasion, and migration of HepG2 cells.

**Fig 3:**
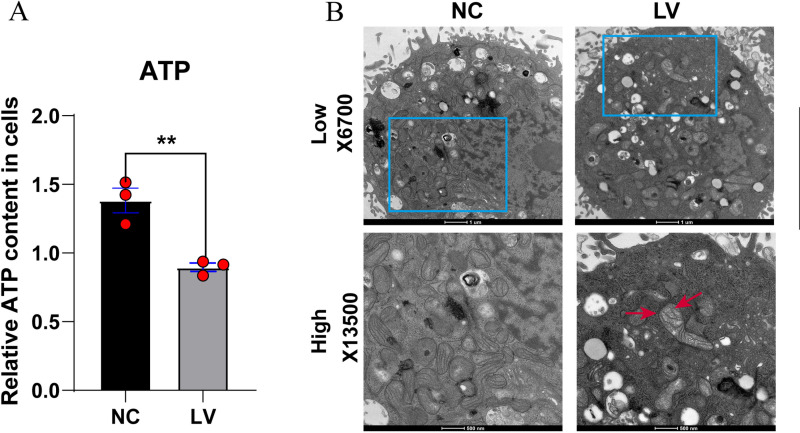
PCYT2 overexpression promotes HepG2 cells mitochondrial damage. **(A)** The ATP levels of each group (NC, normal control; LV, lentivirus transfection to over-express PCYT2) were detected using commercial reagent kits (n = 3 per group). **(B)** Images of mitochondrial damage post-PCYT2 overexpression were captured via transmission electron microscopy. Data are presented as means ± SD. **P < 0.01.

During cell apoptosis, the mitochondria undergoes many changes, such as respiratory chain depolymerization, oxidative phosphorylation, decreased ATP synthesis, and increased reactive oxygen species. We measured the concentration of ATP in the cells of the NC and LV groups (Fig 3A), and photographed the mitochondria using transmission electron microscopy (Fig 3B). The results showed that the LV group exhibited mitochondrial damage and its ATP content was lower than that in the NC group. Compared to the NC group, in the LV group, the mitochondria was swollen and its number was significantly reduced (Fig 3B), further suggesting that PCYT2 overexpression promotes apoptosis.

### CDP-Etn inhibits HepG2 cells proliferation and migration

**Fig 4:**
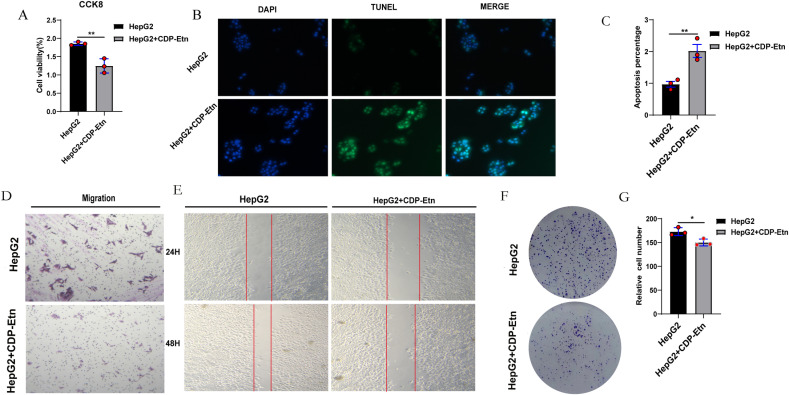
CDP-Etn inhibits the proliferation and migration of HepG2 cells. **(A)** The proliferation ability of HepG2 cells with or without CDP-Etn supplementation were detected using CCK-8 (n = 3 per group). **(B–C)** HepG2 cells underwent TUNEL staining, and the nucleus underwent DAPI staining. Magnification: 100 × **(D–G)** Transwell **(D)**, scratch wound healing (**E**) and colony formation assays (**F–G**) were performed post-CDP-Etn supplementation. Magnification for Transwell and scratch wound healing assay is 100 × . Data are presented as means ± SD. *P < 0.05, **P < 0.01.

PCYT2’s inhibition of cellular proliferation and migration was mediated by altering downstream metabolite levels, CDP-Etn was introduced to HepG2 cells, and cell viability was measured using CCK-8. Therefore, HepG2 + CDP-Etn cells have lower cell viability than HepG2 cells (Fig 4A). In addition, TUNEL staining was performed to observe apoptosis in HepG2 and HepG2 + CDP-Etn cells; the results indicated that CDP-Etn promoted apoptosis in HepG2 cells, affirming those of the CCK-8 assay (Fig 4B and 4C). Furthermore, Transwell assay (Fig 4D) and scratch experiment (Fig 4E) was conducted to detect cell migration, suggesting that supplementation with CDP-Etn inhibited cell migration. Subsequently, plate cloning showed that fewer colonies formed in HepG2 + CDP-Etn cells than in HepG2 cells (Fig 4F and 4G). Thus, we confirm that CDP-Etn inhibited the proliferation and migration ability of HepG2; that is, PCYT2 inhibited proliferation and migration by altering downstream PE metabolism.

### PCYT2 overexpression inhibits tumor growth in vivo

**Fig 5:**
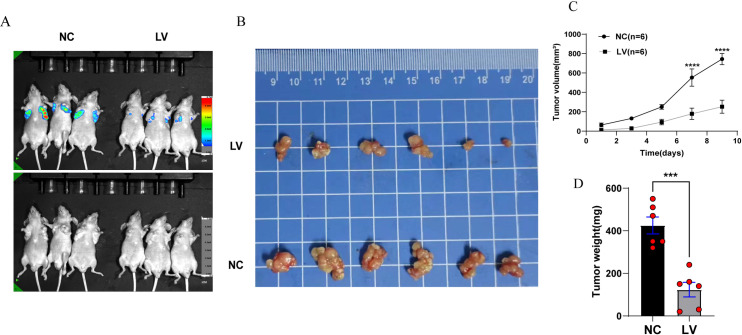
PCYT2 overexpression inhibits tumor growth in vivo. **(A–B)** Representative image of in vivo imaging system (**A**) and tumors isolated from mice xenograft model **(B)**, which were established by subcutaneously implanting PCYT2-overexpressing HepG2 cells (LV) and blank control HepG2 cells (NC). **(C, D)** Cancer volumes were measured every other day, and the average tumor weights in each group were measured. n = 6 per group, data are presented as means ± SD. ***P < 0.001, ****P < 0.0001.

To investigate whether PCYT2 affects hepatocarcinogenesis and development in vivo, we subcutaneously implanted PCYT2-overexpressing HepG2 cells and blank control HepG2 cells into 12 nude BALB/c mice. The tumor volume was measured at 2 d intervals(Fig 5C). After 20 d, the mice were anesthetized with isoflurane to observe the tumor volume using an in vivo imaging system (Fig 5A). The nude mice were sacrificed and tumors were removed for imaging (Fig 5B) and weighing (Fig 5D). The diameter of the largest tumor was 1.9 cm. The results showed that the fluorescence intensity of the tumors in the LV group was weaker than that in the NC group and the tumor volume and weight in the NC group were greater than that in the LV group, suggesting that PCYT2 overexpression inhibit tumor growth in vivo.

## Discussion

PCYT2 is a rate-limiting enzyme in PE synthesis that is commonly used in the study of obesity-related diseases, such as non-alcoholic fatty liver disease and type 2 diabetes [12,20–22]. Recently, interest in the role of PCYT2 in cancer has been growing [18,23,24]. Previous studies show that PCYT2 has different roles in various cancers and cancer settings. For instance, in metastatic colorectal cancer (CRC), PCYT2 is significantly downregulated and functions as a tumor metastasis inhibitor [25]. In human breast cancer cells (MCF-7), the level of PCYT2 in cancer cells is elevated in response to the stressful environment [26].

Our findings show that PCYT2 expression is abnormally downregulated in HepG2 cells, which is consistent with that of previous studies where in PCYT2 expression was downregulated in MCF-7 and invasive human metastatic CRC [16,17]. Although PCYT2 regulates several human cancers [18,19,23], its role in HCC cells remains unknown. Based on the literature and our findings, PCYT2 in human cancers appears to have a consistent expression profile in human cancers, irrespective of tumor origin or location [27].

Regarding the mechanism whereby PCYT2 influences cancer development, a previous report showed that PCYT2 downregulation-induced phosphoethanolamine (PEtn) accumulation correlated with tumor growth under nutrient starvation, thereby PCYT2 overexpression reduced PEtn levels and tumor growth [18]. However, in the present, we found that CDP-Etn supplementation inhibited HCC migration, invasion and proliferation. In our previous study, we showed that the levels of BAX and cleaved caspase-3 were significantly increased whereas Bcl-2 was significantly reduced in the livers of type 2 diabetic mice and L02 cells after stimulation with high glucose and free fatty acids (HG&FFA)(12). CDP-Etn (100 μM) protected cells from HG&FFA-induced apoptosis by reducing BAX and cleaved caspase-3 levels as well as and increasing Bcl-2 levels [12]. Whether PCYT2 downregulation induced PEtn accumulation contributes to HCC development further study.

Increasing evidence suggests that PCYT2 is aberrantly expressed in various models of liver disease and may predict clinical outcomes in patients. PCYT2 is instrumental in the deregulation of these processes leading to the development of obesity, insulin resistance, liver steatosis and dyslipidemia [28]. CDP-Etn supplementation has been reported to alleviate PCYT2 deficiency engendering age-dependent and insulin-resistant non-alcoholic steatohepatitis to improve patient prognosis [20,29–31]. Chronic administration of peroxisome proliferators can increase the content of hepatic PC and PE for hepatomegaly and proliferation as well as cause liver cancer in rodents [32,33]. Based on the current studies, we hypothesized that PCYT2 may be involved in the regulation of cellular processes in HCC. Therefore, we utilized in vivo and in vitro validation methods to assess the expression and mechanistic roles of PCYT2 in liver cancer cells. Herein, PCYT2 overexpression was determined to inhibit HCC cell proliferation, migration and invasion both in vitro and in vivo. And, the number and morphology of mitochondria in HCC cells overexpressing PCYT2 were significantly different from those in HCC cells without any treatment, such as a decrease in the number of mitochondria and swelling of the mitochondria. These changes suggest that PCYT2 affects the mitochondrial function of cells. Notably, when the HepG2 cells received CDP-Etn supplementation, their proliferation, migration, and invasion were inhibited in vitro.

Notably, the ATP level decreased in HCC cells following the overexpression of PCYT2, and the cells were found to be accompanied by mitochondrial damage using transmission electron microscopy. However, previous reports have indicated that PCYT2 is present only in the endoplasmic reticulum of hepatocytes [14,34]. Additionally, the phenotype of liver PCYT2^-/-^ knockout mice showed no signs of liver injury, however, they experienced massive accumulation of liver triglycerides (TAG) [35]. Therefore, we hypothesized that the influence of PCYT2 on mitochondrial function is mediated by metabolites such as TAG, DAG, and PE. However, further studies are needed to clarify whether the PCYT2 exerted inhibition of HCC cells alleviates mitochondrial damage. As understanding, the mechanism whereby PCYT2 overexpression causes mitochondrial damage will deepen our understanding of PCYT2 regulation in HCC cells.

In conclusion, this study provided compelling data demonstrating the aberrant expression and functional role of PCYT2 in HepG2 cells. PCYT2 expression levels were lower in HepG2 than in L02. Furthermore, PCYT2 overexpression in HepG2 cells induced mitochondrial damage; inhibited proliferation, invasion, and migration; and promoted cell apoptosis.

## Supporting information

S1 Fig Cleaved-caspase-3, Caspase-3 protein expression. (**A–B**) The protein expression of Caspase-3 and Cleaved-caspase-3 was measured per group (NC, normal control; LV, lentivirus transfection to over-express PCYT2) using western blotting and representative protein quantification (n = 3 per group).
(TIF)
