# Supplementary figures and images for: PCYT2 overexpression induces mitochondrial damage and promotes apoptosis in hepatocellular carcinoma cells
Source: PLoS One. 2025 May 28;20(5):e0323974. doi: 10.1371/journal.pone.0323974 (PMC12118871; doi:10.1371/journal.pone.0323974)

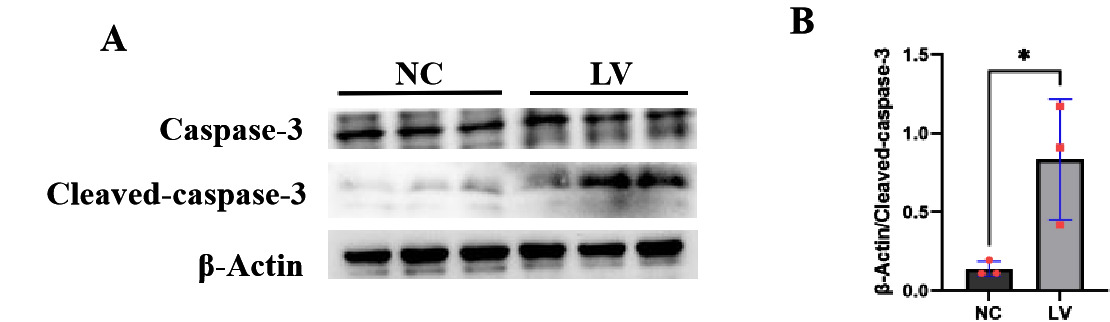

Supplement: S1 Fig — (A–B) The protein expression of Caspase-3 and Cleaved-caspase-3 was measured per group (NC, normal control; LV, lentivirus transfection to over-express PCYT2) using western blotting and representative protein quantification (n = 3 per group). (TIF) [file pone.0323974.s001.tif]
